# Unique Endomembrane Systems and Virulence in Pathogenic Protozoa

**DOI:** 10.3390/life11080822

**Published:** 2021-08-12

**Authors:** Mark F. Wiser

**Affiliations:** Department of Tropical Medicine, Tulane University School of Public Health and Tropical Medicine, New Orleans, LA 70112, USA; wiser@tulane.edu

**Keywords:** protozoa, secretory pathway, endomembranes, pathogenesis, virulence, host-parasite interaction, *Giardia*, kinetoplastids, *Trypanosoma*, Apicomplexa, *Plasmodium*, *Toxoplasma*

## Abstract

Virulence in pathogenic protozoa is often tied to secretory processes such as the expression of adhesins on parasite surfaces or the secretion of proteases to assisted in tissue invasion and other proteins to avoid the immune system. This review is a broad overview of the endomembrane systems of pathogenic protozoa with a focus on *Giardia*, *Trichomonas*, *Entamoeba*, kinetoplastids, and apicomplexans. The focus is on unique features of these protozoa and how these features relate to virulence. In general, the basic elements of the endocytic and exocytic pathways are present in all protozoa. Some of these elements, especially the endosomal compartments, have been repurposed by the various species and quite often the repurposing is associated with virulence. The Apicomplexa exhibit the most unique endomembrane systems. This includes unique secretory organelles that play a central role in interactions between parasite and host and are involved in the invasion of host cells. Furthermore, as intracellular parasites, the apicomplexans extensively modify their host cells through the secretion of proteins and other material into the host cell. This includes a unique targeting motif for proteins destined for the host cell. Most notable among the apicomplexans is the malaria parasite, which extensively modifies and exports numerous proteins into the host erythrocyte. These modifications of the host erythrocyte include the formation of unique membranes and structures in the host erythrocyte cytoplasm and on the erythrocyte membrane. The transport of parasite proteins to the host erythrocyte involves several unique mechanisms and components, as well as the generation of compartments within the erythrocyte that participate in extraparasite trafficking.

## 1. Introduction

Secretion has long been recognized as a virulence factor in pathogen bacteria. Various secretion pathways have been defined and these bacterial secretory pathways are responsible for the secretion of adhesins, invasins, and toxins associated with bacterial pathogenesis [1]. This same concept can also be applied to pathogenic protozoa. For example, pathogenic protozoa express proteins that participate in host-parasite interactions that can be classified as adhesins. Some of these adhesins may assist the pathogen in invading tissues or cells. In addition, many pathogens secrete proteases and other factors that break down tissues and cause inflammation. Obviously, adhesins and other virulence factors must be expressed at the interface between the pathogen and the host, such as a cell surface location or secretion into the host. Proteins expressed on the surface of cells typically have signal sequences that direct proteins to the cell surface via the endoplasmic reticulum (ER) and Golgi apparatus. Similarly, secreted virulence factors are also processed by these same endomembrane systems.

Most of our knowledge on endomembrane systems is derived from yeast and mammalian systems and in general the endomembranes are not well characterized in protozoa. All protozoa have the basic elements of the eukaryotic secretory pathway and endomembrane system. However, variations in the endomembrane system of protozoa have been noted and include the loss of some organelles or the acquisition of unique organelles that are only found in specific phylogenic groups [2]. Often these variations in endomembranes play a role in disease pathogenesis. This review provides a broad overview of endomembrane systems and secretion of virulence factors in pathogenic protozoa. In particular, the uniqueness of endocytic and exocytic processes in pathogenic protozoa and how these processes contribute to pathogenesis is highlighted.

Protozoa exhibit an extreme range of diversity and do not represent a monophyletic group [3]. Organisms that are typically viewed as protozoa are found in all the major eukaryotic supergroups (TSAR—an acronym for telonemids, stramenopiles, alveolates, and rhizaria, Haptista, Cryptista, Archaeplastida, Amorphea, CRuMs—an acronym for collodictyonids, Rigifilida, and *Mantamonas*, Discoba, Metamonada, Hemimastigophora, and Excavata) [4]. Several species from these various supergroups are human pathogens and a few species are capable of causing severe human disease [5,6]. The most common pathogenic protozoa are *Giardia duodenalis* (giardiasis), *Entamoeba histolytica* (amebic dysentery), *Trichomonas vaginalis* (trichomoniasis), *Trypanosoma brucei gambiense* (African sleeping sickness), *Trypanosoma cruzi* (Chagas disease), *Leishmania* species (leishmaniasis), *Plasmodium* species (malaria), *Toxoplasma gondii* (toxoplasmosis), and *Cryptosporidium* species (cryptosporidiosis). These species and diseases are the focus of this review due to their medical importance, and the fact that more work has been done on these species as compared to less virulent protozoa.

## 2. Basic Endomembrane Systems

**Figure 1:**
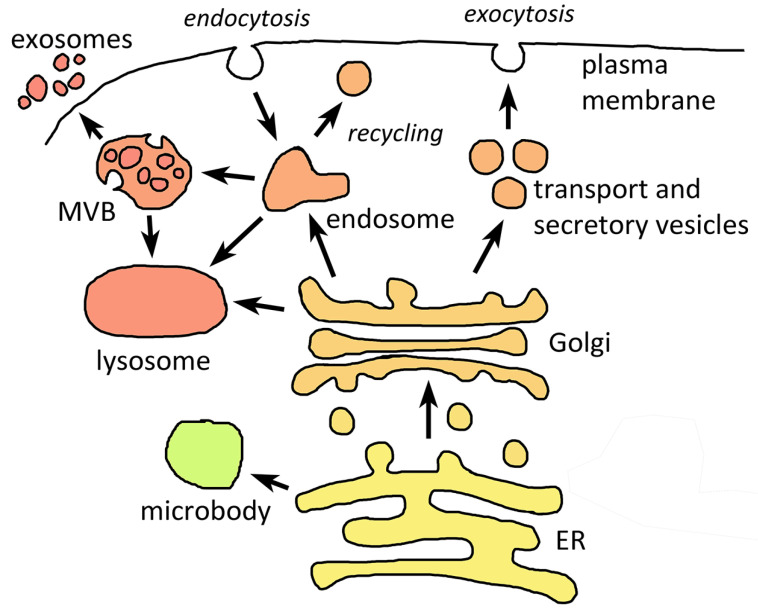
Major components of eukaryotic endomembrane systems that are present in protozoa. The endoplasmic reticulum (ER) plays a central role in the generation of the endomembranes of eukaryotic cells. Vesicles shuttle membrane components and proteins between the ER and the various endomembranes such as Golgi and microsomes. Endocytosed material is transported to lysosomes or multivesicular bodies (MVB) via the endosome. MVB can be incorporated into the lysosome and degraded or secreted from the cell as extracellular vesicles called exosomes.

Phagocytosis and the development of endomembrane systems and membrane trafficking is paramount in the origin of eukaryotes [7,8], and therefore, it is expected that pathogenic protozoa would contain similar endomembranes as other eukaryotes. Further, and indeed, the key components of endocytic and exocytic pathways are present in all protozoa. Key components of the endomembrane system include the nuclear envelope, endoplasmic reticulum (ER), Golgi, lysosomes and related vacuoles, endosomes, microbodies, vesicles that transport material between compartments, and the plasma membrane (Figure 1). Proteins destined to be secreted or transported to organelles typically have a signal sequence and pass through the ER and Golgi via transport vesicles [9]. However, the secretomes of pathogenic protozoa include many proteins being exported by unconventional protein secretion [10].

The ER is a membrane network that emanates from the outer membrane of the nuclear envelope. The function of the ER includes lipid synthesis, the first step in protein secretion and sorting, and quality control of protein folding. Membrane networks corresponding to the ER have been identified in all protozoa, and for the most part, the functions of the ER are preserved. Although, in regards to quality control and ER stress, the elements of ER-associated degradation and unfolded protein response do differ slightly between the pathogenic protozoa and other eukaryotes [11]. Proteins destined for the plasma membrane, other organelles, or to be secreted are packaged into vesicles at ER exit sites and are usually transported to the Golgi. The Golgi consists of flattened membrane disks, called cisternae. The cisternal face proximal to the ER is the cis-Golgi network and the face on the opposite side is the trans-Golgi network. Some pathogenic protozoa do not have the iconic stacks of Golgi cisternae (Table 1). Evolutionarily, this is likely due to a reduction of the Golgi and its function, and this reduction has occurred independently several times during evolution [12].

The secretory pathway is also linked with endocytosis and lysosomes [30]. Many lysosomal proteins are synthesized in the ER and subsequently trafficked to the lysosome via the Golgi and trans-Golgi network. The endosomes contain material taken up via endocytosis and fuse with lysosomes where the endocytosed material is degraded. Plasma membrane components are returned to the plasma membrane (Figure 1). In some protozoa the lysosomes function as food vacuoles where the digestion of macromolecules takes place, and overall, these lysosomes are similar to other eukaryotes. Sometimes the details vary due to specialized functions. For example, the food vacuole of the malaria parasite during the blood stage infection is highly specialized for the digestion of hemoglobin [29].

Microbodies have been described in some of the pathogenic protozoa [25]. Microbodies are more commonly known as peroxisomes since quite often they are involved in hydrogen peroxide mediated fatty acid oxidation. However, there is a wide diversity of enzyme content and metabolic functions across eukaryotes and that includes pathogenic protozoa (Table 1). Notable are the glycosomes of the kinetoplastids in which the enzymes of glycolysis are localized [20]. In addition, also notable is the apparent lack of microbodies in *Giardia*, *Trichomonas*, *Entamoeba*, *Plasmodium*, and *Cryptosporidium* [25]. Microbodies are directly derived from the ER and import of proteins into microbodies involves components that are homologous to components of the ER-associated protein degradation pathway [31].

## 3. Extracellular Vesicles

Extracellular vesicles (ECV) are membrane-bound particles released from cells. They have been described in bacteria, fungi, protozoa, plants, and animals [32]. Generally, two major classes of ECV are recognized: exosomes and ectosomes [33,34]. Exosomes originate from the endocytic pathway via the formation of multivesicular bodies (MVBs) which fuse with the plasma membrane to release the exosomes. The MVBs are often part of autophagocytosis. Ectosomes, also called microvesicles, form from a protrusion of the plasma membrane resembling the budding of enveloped viruses from cells.

In pathogenic protozoa exosomes are the more prominent of the two [35,36,37]. In the case of intracellular pathogens, the ECV can be derived from either the parasite or the host cell [38]. Little work has been done on the biogenesis of ECV in pathogenic protozoa and it is presumed that the biogenesis is similar to other eukaryotes. The formation of the MVB generally involves an inward invagination of the endosomal membrane and nanovesicles accumulate within the endosome [30]. Generally, the MVB fuse with the lysosomes and their contents are degraded. However, if the MVB fuses with the plasma membrane, the ECV are released into the extracellular milieu (Figure 1). ECV formation in African trypanosomes involves a different process in which the ECV vesiculate from tubular structures called nanotubes that originate from the flagellar membrane [39].

### Extracellular Vesicles Are Virulence Factors in Pathogenic Protozoa

A wide variety of metabolites, lipids, proteins, and nucleic acids has been identified as cargo in the ECV, and accordingly, a wide range of functions have been ascribed to the ECV. Such functions include roles in parasite-to-parasite communication and differentiation processes [35,36]. For example, exosomes promote sexual differentiation in the malaria parasite [40]. ECV often play a role in disease pathogenesis by facilitating parasite growth and survival, as well as, promoting inflammation and damage to the host [41]. Specific examples of ECV as virulence factors include enhancing adherence, inducing inflammation, and evading immune responses [35,36].

## 4. Anaerobic Luminal Pathogens

*Giardia*, *Trichomonas*, and *Entamoeba* are quite distinct in terms of evolution and are not closely related phylogenetically. *Giardia, Trichomonas*, and the kinetoplastids are all in the super group Excavata. However, questions have been raised about whether Excavata is truly a phylogenetic group [4]. *Giardia*, *Trichomonas*, and *Entamoeba* do exhibit similarities in that they parasitize lumens and exhibit anaerobic metabolisms. In addition, all three possess a mitochondrion-related organelle associated with anaerobic metabolism [16]. In *Giardia* and *Entamoeba* this reduced mitochondrion is called the mitosome and in *Trichomonas* it is called the hydrogenosome (Table 1). A unique endosomal pathway for the targeting of proteins to the hydrogenosome has been proposed [42].

### 4.1. Pathogenesis Associated with Luminal Pathogens Is Associated with Cytoadherence and Secreted Proteases

Another similarity between all three pathogens is that they adhere to an epithelium, and this adherence plays a role in their pathogenicity. *Giardia* adheres to the small intestine via a structure called the adhesive disk [43]. Various adhesins have been described from *Entamoeba* [44], and in particular, the Gal/GalNAc lectin is involved in the contact dependent killing of epithelial cells in the large intestine [45]. Similarly, several adhesins from *Trichomonas* that participate in the adherence to the urogenital tract have been described [46]. In addition, all three pathogens secrete factors, such as proteases, that have pathological effects on the epithelium including cytotoxicity [47,48,49,50]. Thus, secretory processes contribute to virulence. However, not much specific work has been carried out on the trafficking of these membrane proteins and secreted factors.

### 4.2. Giardia Appears to Lack a Golgi and a Conventional Lysosome

The secretory pathways of *Giardia*, *Trichomonas*, and *Entamoeba* have not been extensively characterized. The morphology of the ER/Golgi in *Trichomonas* is similar to the typical eukaryotic secretory pathway and the overall function appears the same [17]. A stacked Golgi is not seen in *Entamoeba*, but nonetheless, its secretory pathway is similar to those of other eukaryotic cells [18,51,52]. A stacked Golgi is also not observed in *Giardia*. In this case though many of the normal Golgi functions are greatly reduced and the sorting functions of the Golgi appear to be carried out by the ER [13,53]. However, during encystation the parasite secretes a large amount of cell-wall material via encystation-specific vesicles and these encystation-specific vesicles do exhibit some features reminiscent of the Golgi [54]. In addition, the only Rho GTPase of *Giardia* coordinates cyst wall protein trafficking [55]. Some Rho GTPases are associated with the Golgi and likely play roles in regulating intracellular trafficking [56].

Peripheral vacuoles located below the plasma membrane are seen in *Giardia* [15]. These vacuoles have characteristics of both lysosomes and endosomes, and they appear to be the only endocytic organelle of *Giardia* [57]. In addition, peripheral vacuoles are also involved in the trafficking of plasma membrane proteins and secreted soluble proteins [13,53]. This trafficking of proteins to the plasma membrane via the peripheral vacuoles is distinct from the trafficking of the major plasma membrane protein called variant surface protein. Further, both of these pathways are distinct from the encystation-specific vesicles.

## 5. Kinetoplastids

Kinetoplastids are a monophyletic group originally identified by the presence of concatenated mitochondrial DNA that forms a distinct staining structure called the kinetoplast [58]. Human disease caused by kinetoplastids include human African trypanosomiasis (*Trypanosoma brucei gambiense* and *T. b. rhodesiense*), Chagas disease (*T. cruzi*), and leishmaniasis (several *Leishmania* species). African trypanosomes do not secrete significant levels of macromolecules, whereas *T. cruzi* and *Leishmania* secrete virulence factors that assist in their survival within macrophages and other host cells [59,60,61]. In the case of *Leishmania*, the parasite is taken up by phagocytosis and the parasite only infects professional phagocytes. Following fusion of the phagosome fuses with the lysosome, the parasite secretes factors that shut down the lysosome functions and allow the parasite to survive. *T. cruzi* is also an intracellular parasite that relies on host cell phagocytosis to gain entry. However, *T. cruzi* is also capable of infecting non-phagocytic cells via the induction of membrane repair in the host cell [62]. This results in the recruitment of lysosomes to the parasite attachment site followed by an incorporation of the parasite into compartment analogous to the phagosome. In contrast to *Leishmania*, *T. cruzi* secretes factors that allow it to escape from the phagosome [63].

### 5.1. The Flagellar Pocket Is the Primary Site of Endocytosis and Exocytosis in Kinetoplastids

**Figure 2:**
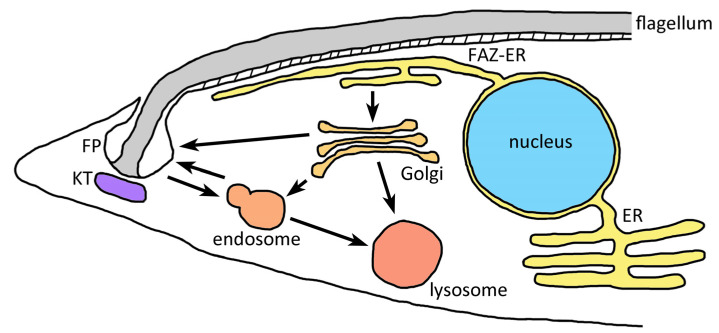
Polarized membrane trafficking in kinetoplastids. The flagellar pocket is an invagination of the plasma membrane at the base of the flagellum near the kinetoplast (KT). The Golgi, endosomes, and lysosomes are also located in the vicinity of the flagellar pocket (FP). A specialized region of the ER called the flagellar adherence zone ER (FAZ-ER) is also part of the polarized membrane trafficking. Figure modeled after Field and Carrington [65].

Most of the work on membrane trafficking in the kinetoplastids has been carried out in the African trypanosomes and especially *T. b. brucei*. Overall, membrane trafficking in the kinetoplastids is similar to other eukaryotes [19,64,65]. No particularly extraordinary endomembranes have been noted. One unique feature of the kinetoplastids is that the membrane trafficking at the plasma membrane is primarily restricted to the flagellar pocket [21]. The flagellar pocket is an invagination of the plasma membrane where the flagellum emerges from the cell. Restriction of endocytosis and exocytosis to the region around the flagellar pocket results in a polarization of the Golgi, endosomes, and lysosome to a region near the flagellar pocket (Figure 2). As part of this polarization an extension of the ER is located just under the plasma membrane where the flagellum attaches to the parasite surface [66]. This region of the plasma membrane is called the flagellar adherence zone (FAZ). Presumably this FAZ-ER specializes in membrane trafficking involved in secretion and endocytosis.

### 5.2. Glycosylphosphatidylinositol Anchors Are Abundant on the Plasma Membranes of Kinetoplastids

Another unique feature of the kinetoplastids is the predominance of glycosylphosphatidylinositol (GPI)-linked proteins and glycolipids on their cell surfaces [19,67]. In fact, GPI anchors were first described in the African trypanosomes. The GPI-anchored proteins and lipids are important virulence factors in all three human kinetoplastids. Specifically, GPI anchors participate in the formation of dense protective coats on the parasite surfaces. In the case of African trypanosomes, a single GPI-anchored protein called variant surface glycoprotein (VSG) constitutes approximately 90% of the surface of bloodstream trypomastigote forms. An exceptionally high rate of endocytosis has been noted for the blood stage of African trypanosomes. It has been proposed that this high rate of endocytosis is involved in the removal and degradation of antibodies bound to VSG [19]. Following endocytosis, VSG is recycled back to the plasma membrane.

Predominant GPI-anchored proteins on the surface of *T. cruzi* include mucin-like glycoproteins and an associated trans-sialidase which play critical roles in parasite adherence and immune evasion [68]. The predominant GPI-anchored virulence factors of *Leishmania* are a metalloprotease, called GP63, and lipophosphoglycan (LPG). Interestingly, GP63 and LPG are shed from the parasite surface after phagocytosis by macrophages and are redistributed in the host cell via the secretory pathway of the host cell [69]. GPI-anchored proteins are also well represented in the Apicomplexa and participate in the immunopathology associated with malaria and toxoplasmosis [70].

## 6. Apicomplexa and Apical Organelles

Most apicomplexans spend at least a portion of their life cycle as intracellular parasites. In contrast to *Leishmania* and *T. cruzi*, which depend on host endocytic mechanisms to gain entry into host cells [71,72], the apicomplexans have evolved a rather sophisticated mechanism involving specialized secretory organelles called micronemes and rhoptries (Table 2). These organelles are located on one end of the parasite and are associated with cytoskeletal elements to from the apical complex. The apical complex is one of the key attributes of this phylogenetic group and the basis for the name Apicomplexa. Early on it was surmised that these apical organelles participate in the invasion process since most apicomplexans enter cells from the apical end. Further, and indeed, the secretion of the contents of micronemes and rhoptries plays a key role in host cell invasion [22,73]. In addition, as the parasite invades the host cell a parasitophorous vacuole is formed. This vacuole superficially resembles the phagosome in that a membrane, called the parasitophorous vacuolar membrane (PVM), surrounds the parasite. However, this PVM is primarily generated by the parasite and does not represent an endocytic compartment.

### 6.1. Micronemes and Rhoptries Facilitate Host Cell Entry, Exit and Modification

Exocytosis of the micronemes releases adhesins, proteases, and perforins that play crucial roles in host cell invasion and egress, gliding motility, and migration across biological barriers [74,75]. During host cell invasion the release of the microneme contents occurs immediately after the parasite makes contact with the host cell. Fusion of the microneme to the plasma membrane exposes adhesins on the parasite surface that bind to receptors on the surface of the host cell resulting in the formation of a junction between the parasite and host cell [22]. The cytoplasmic domains of the transmembrane adhesins interact with a multi-protein complex called the glideosome [76]. The glideosome contains actino-myosin motor proteins that generate the force involved in gliding motility and parasite entry into host cells. The junction formed between the host cell and parasite is pulled towards the posterior of the parasite by the glideosome and is called a moving junction. Movement of this junction towards the posterior end of the parasite propels the parasite forward into the host cell.

The rhoptries are club-shaped membrane bound organelles with a duct at the apical end of the parasite. As the parasite enters the host cell, the contents of the rhoptries are discharged [77]. The rhoptry is divided into the neck region and the bulb region and different proteins with different functions segregate to these different sub-compartments of the rhoptry [77,78]. Proteins from the rhoptry neck are required for invasion and participate in the formation of the glideosome and moving junction [77]. The bulb of the rhoptry contains membranous material that participates in the formation of the parasitophorous vacuolar membrane (PVM) as well as modifying the parasitophorous vacuole [79].

### 6.2. Dense Granules Participate in the Modification of Host Cells by Apicomplexa

Following the completion of invasion, the contents of the dense granules are secreted into the parasitophorous vacuole [80]. Cargo proteins of the dense granules do not participate in the invasion process, but rather modify the parasitophorous vacuole and the host cell. In *Plasmodium* all the dense granules are discharged immediately after invasion and are not regenerated again until merozoites are reformed during parasite replication. In other apicomplexan species, dense granules are produced and discharged throughout the intracellular period [81]. In those species there are likely two populations of dense granules. One population of dense granules, like those in *Plasmodium*, are generated during the production of invasive forms, and these dense granules are discharged soon after invasion of a new host cell. The other population of dense granules are secretory vesicles that are continuously produced and discharged as the intracellular parasite grows within the host cell.

### 6.3. The PVM Is an Interface between the Parasite and the Host Cell

During cell invasion the parasite generates the PVM that surrounds the parasite, and many apicomplexans reside in the parasitophorous vacuole throughout their intracellular development. Some apicomplexans, such as *Babesia* [82], escape from the vacuole and reside in direct contact with the host cell cytoplasm. Initially, the lipid bilayer of the PVM is primarily derived from the host cell plasma membrane during invasion [83,84]. Host membrane proteins are largely excluded from the PVM during invasion and most proteins of the PVM are of parasite origin. In addition, as the parasite grows, the PVM expands through the addition of material from the parasite. The PVM does potentially offer some survival benefits in some situations since lysosomes cannot fuse with the parasitophorous vacuole. Thus, the PVM also serves as a barrier between the host and parasite. Since the intracellular parasite acquires nutrients directly form the host, the PVM possesses parasite derived channels that are permeable to metabolites.

### 6.4. Endosomal Pathways May Function in the Formation of Micronemes and Rhoptries

The micronemes and rhoptries are generated de novo during parasite replication and the formation of invasive stages. These apical organelles are likely generated post-Golgi and involve an endosome-like compartment [85,86]. Thus, it appears that apicomplexans have repurposed components of the endosomal system as intermediate compartments for the generation of micronemes and rhoptries [87]. In particular, decreased expression of sortilin, a Golgi protein involved in transporting proteins to the endosomal pathway, adversely affects the generation of micronemes and rhoptries and merozoite formation [88,89]. The function of sortilin may be to escort apical organelle proteins to their final destinations [90]. Knockdown of sortilin expression in *P. falciparum* also disrupts the biogenesis of dense granules [89] suggesting that the endosomal-like compartment is involved in protein sorting to the dense granules as well as the micronemes and rhoptries. In addition, positioning of these post-Golgi compartments and the apical organelles may involve an unconventional myosin motor [91].

### 6.5. Myzocytosis and the Apical Organelles

Not all apicomplexans are intracellular pathogens, but nonetheless have apical organelles. For example, many gregarines—parasites primarily of annelids and insects—attach to host cells and the apical organelles mediate these host-parasite interactions [92]. A similar type of interaction is also seen in predatory flagellates called colpodellids [93]. *Colpodella* and related species attach directly to their prey at the apical end and discharge the contents of the micronemes and rhoptries into the prey cell. At the attachment site the membrane of the prey is disrupted so that the plasma membrane of the predatory colpodellid is in direct contact with the cytoplasm of the prey cell. Subsequently, the cytoplasm of the prey cell is taken up by pinocytosis, transported to a food vacuole, and digested. This type of feeding is called myzocytosis. A rhoptry protein of the Apicomplexa is conserved in *Colpodella* [94]. This suggests some commonality between the junction formed between predator and prey during myzocytotic feeding and the moving junction form during host cell invasion.

*Cryptosporidium* appears to occupy a position between the myzocytotic feeding of the gregarines and colpodellids and the intracellular parasitism exhibited by many apicomplexans. *Cryptosporidium* sporozoites and merozoites attach to intestinal epithelial cells at their apical ends and the discharge of the apical organelles mediates the formation of a junction between the host and parasite [93,95]. Nutrients are transported from the host cell to the parasite via this junction called the feeder organelle [96]. Distinct from the colpodellids and gregarines, the nutrients are taken up via membrane transporters instead of pinocytosis [97]. In addition, *Cryptosporidium* remodels the host cell actin and causes an expansion and fusion of the membranes of the microvilli and these remodeled microvilli surround and enclose the parasite [98]. This location is referred to as extracytoplasmic since the parasite is not inside of the host cell, but at the same time, the parasite is enclosed by a membrane of host cell origin.

The original function of the apical organelles was likely involved in feeding via myzocytosis. These organelles predate the Apicomplexa, in that similar organelles are found in predatory dinoflagellates and perkinsids that also exhibit myzocytotic feeding [93]. Dinoflagellates and apicomplexans are sister groups within Alveolata [99]. Furthermore, parallels between the rhoptries of apicomplexans and the trichocysts of predatory ciliates have been made [100]. The trichocysts are secretory organelles that are discharged upon contact with prey organisms [101]. This discharge immobilizes the prey and allows for their endocytosis and digestion. In a switch from a predatory lifestyle to a parasitic lifestyle, the apical organelles were likely repurposed to facilitate entry into host cells.

## 7. Alveolates and the Inner Membrane Complex

Another unique endomembrane compartment found in Apicomplexa is the inner membrane complex (IMC). Compartments analogous to the inner membrane complex are also found in ciliates, dinoflagellates, and some related genera, such as *Perkinsus*, *Colpodella*, and *Colponema* [102]. Although these three major groups are rather distinct, they all are characterized by membrane-bound vesicles and associated proteins that lie just under the plasma membrane. In general, these membrane-bound vesicles are called the cortical alveoli and these structures are the basis for the name for the group. The alveoli have structural roles in determining cell shape, as well as specific roles in the various species [103].

Ciliates and dinoflagellates are primarily free-living organisms found in aquatic environments with relatively few parasitic and pathogenic species. The alveoli of ciliates are flattened submembrane vesicles that are calcium stores with properties similar to the ER [104]. A possible function of these cortical alveoli is the triggering the discharge of trichocysts [101]. In dinoflagellates the cortical alveoli are called amphiesma and may participate in the formation the pellicle [105]. Virtually nothing is known about the biogenesis of alveoli in ciliates and dinoflagellates.

The Inner Membrane Complex Is Derived Largely from the ER

The IMC of Apicomplexa consists of flattened vesicles found in the invasive stages [23]. This creates the appearance of a three-layered membrane pellicle. Coincident with its presence in invasive stages, the IMC and the associated subpellicular microtubules support the glideosome [76]. In *Plasmodium* the IMC is degraded following invasion of host cells or tissues and then regenerated with the production of invasive stages [106]. Biogenesis of the IMC starts at the apical end and progresses down the length of the parasite during the formation of merozoites [103,107] and sporozoites [28]. Thus, the malaria parasite undergoes cycles of IMC generation followed by degradation throughout its complex life cycle [106]. An integral membrane protein of the IMC that is associated with the glideosome redistributes from the ER to the IMC as merozoites are formed [108]. Palmitoylation appears to be involved in the association of other IMC proteins with the IMC [109].

The generation of the IMC in *Toxoplasma* is slightly different than *Plasmodium* in that recycling of IMC material occurs [110]. The differences may relate to the replication process of *Toxoplasma* and how it differs from *Plasmodium*. *Toxoplasma* replicates by a process called endodyogeny in which daughter cells are assembled within the mother cell [111]. In the early stages of daughter cell formation the IMC is generated from the ER as in *Plasmodium* [110]. As replication proceeds IMC material from the mother cell is recycled into the IMC of the daughter cells. This recycling may involve fusion of the maternal IMC with the ER and subsequent movement of IMC material to the daughter IMC via the Golgi and an endosome-like compartment.

## 8. Host Targeting Sequences in Apicomplexa

**Figure 3:**
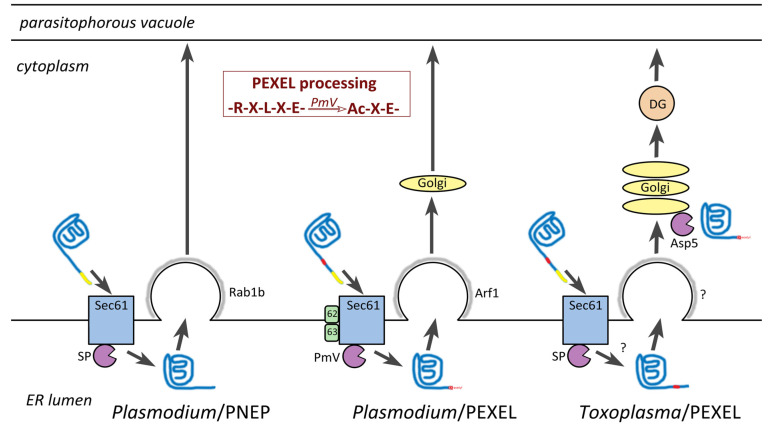
Processing of PEXEL-containing proteins. Exported proteins generally have a hydrophobic signal sequence at their N-terminus (yellow). Some proteins also have the PEXEL motif (red) downstream from the signal sequence. PEXEL-negative exported proteins (PNEP) are translocated into the ER by the canonical Sec61 translocation complex and the signal sequence is removed by signal peptidase (SP). The ER exit sites for these proteins is associated with Rab1b and the proteins are likely transported directly to the parasitophorous vacuole. The translocon for PEXEL-containing proteins is Sec61 complexed with other proteins (Sec62 and Sec63) and the processing is carried out by plasmepsin V (PmV). The inset shows the proteolysis and acetylation (Ac) of the PEXEL motif. Exit from the ER involves Arf1 and proteins probably pass through the Golgi on their way to the parasitophorous vacuole. The details on the entry and exit of *Toxoplasma* export element (TEXEL)-containing proteins has not yet been determined as denoted by the question marks (?), but it is proposed that Sec61 and signal peptidase are involved [114]. Processed TEXEL-containing proteins then move to the Golgi and are further processed at the TEXEL motif by aspartyl protease 5 (Asp5). These processed proteins are then incorporated into dense granules (DG) and carried to the parasitophorous vacuole.

Many apicomplexan proteins that are exported into their host cells contain a unique targeting sequence. This targeting sequence was first identified in proteins exported into the host erythrocyte by the malaria parasite and called the *Plasmodium* export element (PEXEL). PEXEL is typically located downstream of a canonical ER signal sequence and is defined as RxLxE/Q/D [112] where x is any amino acid and the fifth position is either glutamate, glutamine, or aspartate. Within the ER an aspartyl protease, called plasmepsin V, cleaves the PEXEL motif between the third (L) and fourth (x) residues (Figure 3). Following this proteolytic processing the PEXEL-containing protein is acetylated on the N-terminus by an unknown transferase. The recognition, translocation, and proteolytic processing of these PEXEL containing proteins utilize the Sec61 translocation channel on the ER membrane, as do other secreted proteins. However, additional auxiliary proteins are associated with the Sec61 channels that translocate PEXEL-containing proteins [113]. For example, the plasmepsin V is associated with these specialized Sec61 translocation channels instead of the canonical signal peptidase. Other prominent auxiliary proteins are designated as Sec62 and Sec63.

Numerous *Plasmodium* proteins that do not contain PEXEL are also exported into the host erythrocyte and these are generally known as PEXEL-negative exported proteins (PNEPs) [115]. Presumably these PNEPs are translocated into the ER by the conventional Sec61 channel and signal peptidase without the associated Sec62/Sec63. Exit of PEXEL-containing proteins and PNEPs from the ER appears to occur in different subdomains of the ER [116]. In particular, exit of PEXEL-containing proteins are associated with an Arf1 homolog and presumably move from the ER to the Golgi. PNEPs are associated with a Rab1b homolog and presumably are transported directly to the parasitophorous vacuole.

### 8.1. PEXEL-Like Motifs in other Apicomplexans

PEXEL-like motifs (PLM) and orthologs of plasmepsin V have also been identified in *Toxoplasma*, *Babesia*, and *Cryptosporidium* [114,117]. Both *Toxoplasma* and *Babesia* contain a significant number of secreted proteins with a PEXEL-like sequence (eg., RxLx, RxxL, RxL) downstream of a signal peptide. Only 15 proteins with a PLM were identified in *Cryptosporidium*, and in general, the parasite does not appear to secrete many proteins into the host cell. This may reflect the parasite residing in an extracytoplasmic compartment instead of being intracellular. Surprisingly, no PLM were identified in *Theileria* considering the close evolutionary relationship between *Babesia* and *Theileria* [118].

PLM-containing proteins of *Toxoplasma* and *Babesia* proteins are primarily found in the dense granules [117,119,120]. This may not be a major functional distinction since dense granules ultimately secrete their contents into the parasitophorous vacuole and host cell. In addition, RESA, a dense granule protein of *Plasmodium* transferred to the erythrocyte membrane shortly after invasion, has a relaxed PEXEL motif that is processed by plasmepsin V [121]. Dense granules have been identified in *Cryptosporidium* [122], although, little is known about their role on host-parasite interactions. Another distinction between *Toxoplasma* and *Plasmodium* is the processing of the PLM. The processing of the *Toxoplasma* PLM occurs in the Golgi [123] instead of the ER (Figure 3). It is speculated that the signal peptide is removed in the ER before the processing of the PLM in the Golgi. The cellular locations of plasmepsin V orthologs from other apicomplexans is not known.

### 8.2. Possible Ancient Origin of the PEXEL-Like Motif

The presence of a PLM in *Cryptosporidium* suggests PEXEL-based protein targeting has deep roots in the Apicomplexa. *Cryptosporidium* is more closely related to the gregarines than other Apicomplexa and represents an early branch in the apicomplexan tree [92]. Furthermore, PEXEL actually predates the Apicomplexa in that a PLM is also present in the potato pathogen *Phytophthora infestans* [124] and other oomycetes [125,126]. Oomycetes are stramenopiles which form a sister group with the alveolates [4] and possibly diverged from the alveolates in the early Mesoproterozoic period 1.6 billion years ago [127]. Despite this large evolutionary difference, the oomycete PLM is functional in *Plasmodium* [128] and PEXEL is functional in *P. infestans* [129]. In addition, the oomycete PLM is likely subjected to similar proteolytic processing and acetylation [130].

*P. infestans*, the causative agent of the Irish potato famine, and other oomycetes are extracellular pathogens that intimately interact with the host plant cell via a structure called the haustorium [131]. Haustoria are intercellular hyphae that project into the parasitized plant cell. The wall of the plant cell is disrupted, but the plasma membrane of the plant cell remains intact. This forms an intimate interaction between the membranes of the pathogen and host, and in some ways this interaction resembles the relationship between of the plasma membrane of apicomplexan parasites and the PVM. Furthermore, pathogen proteins are translocated across the haustorium membrane and plant cell membrane via a mechanism utilizing the PLM. Factors secreted from the oomycete into the host plant cell suppress plant cell immunity, and thus, a major function of the PLM both in stramenopiles and apicomplexans is the translocation of virulence factors and other proteins into their host cells.

## 9. Remodeling the Host Erythrocyte by the Malaria Parasite

The malaria parasite extensively alters the host erythrocyte during blood-stage merogony [132,133]. These extensive renovations of the host cell are necessary in part due to the rather limited functionality of the erythrocyte. During development, the mammalian erythrocyte loses all organelles and endomembrane systems. In addition, the erythrocyte has a rather scaled-down metabolism. Therefore, the intracellular parasite cannot completely rely upon the host cell for a source of metabolites. To compensate for the paucity of metabolites, new permeability pathways (NPP) are found on the erythrocyte membrane of infected cells [134]. The exact nature of the NPP is not known but parasite proteins that affect the host erythrocyte membrane are clearly involved in the formation of the NPP. Some of the proteins involved in the formation of these NPP originate in the rhoptries and are deposited in the PVM during invasion [135,136]. Subsequently, the proteins are translocated from the PVM to the erythrocyte membrane. The parasite also secretes many other proteins into the host erythrocyte, and in fact, *P. falciparum* may export approximately 10% of its proteome into the erythrocyte [137].

**Figure 4:**
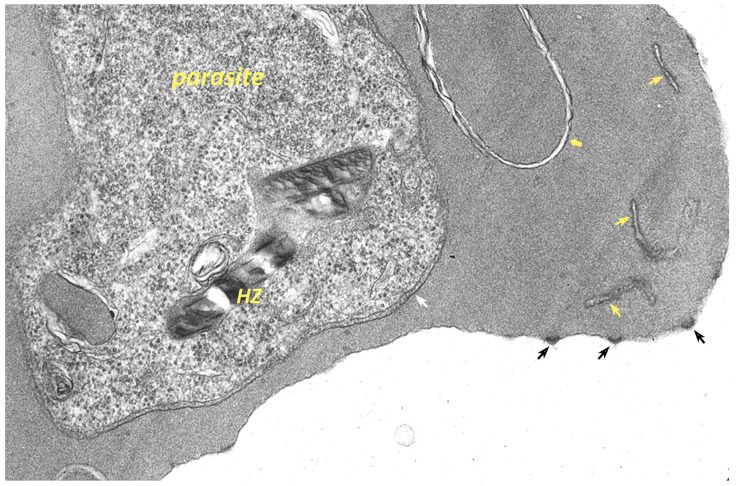
Ultrastructural alterations of the host erythrocyte by *Plasmodium falciparum*. The malaria parasite induces many changes in the host erythrocyte including electron-dense knobs on the erythrocyte surface (black arrows), membranous whorls (yellow block arrow), and Mauer’s clefts (yellow arrows) in the host cell cytoplasm. The parasite is surround by a parasitophorous membrane (white arrow). In addition, also visible is a large hemozoin (HZ) crystal within the parasite. Electron micrograph provided by H. Norbert Lanners.

The malaria parasite also induces numerous ultrastructural alterations in the infected erythrocyte (Figure 4). For example, electron-dense knobs are observed on the surface of *P. falciparum* infected erythrocytes [138] and caveole-vesicle complexes are observed on the surface of *P. vivax* infected erythrocytes [139]. In addition, various membranous tubules and whorls originating from the PVM are also seen in the cytoplasm of the infected erythrocyte. Although best described in *P. falciparum*, such membranous structures are seen in other *Plasmodium* species including rodent malaria parasites [140]. These various membranous extensions of the PVM are sometimes called the tubovesicular network (TVN) [84]. The appearance of the TVN is somewhat variable and sometimes is not present, and therefore, the role of the TVN is not clear. It has been hypothesized that the TVN may be storage site for improperly folded exported proteins [141] and this could explain the variable size and appearance of the TVN. If true, this implies that the TVN is analogous to the ER stress response [11]. Another membrane-bound compartment found in the cytoplasm of *P. falciparum* infected erythrocytes are the Maurer’s clefts [142]. Maurer’s clefts are distinct from the TVN since the two structures have distinct protein compositions.

A particularly well characterized modification of the host erythrocyte by the malaria parasite are the knobs on the surface of *P. falciparum* infected erythrocytes. Proteins synthesized by the parasite and exported to the erythrocyte membrane rearrange the submembrane cytoskeleton of the erythrocyte to form the knobs [133,143]. Embedded in this knob is another parasite protein called erythrocyte membrane protein 1 (*Pf*EMP1). *Pf*EMP1 spans the erythrocyte membrane and is exposed on the erythrocyte surface where it functions as a ligand that binds to receptors on endothelial cells [144,145]. Thus, the knobs serve as focal points for binding of infected erythrocytes to endothelial cells that result in the sequestration of infected erythrocytes in the deep tissues. This sequestration avoids removal by the spleen and promotes parasite survival, and thus, sequestration plays a major role in the increased virulence of *P. falciparum*. Furthermore, sequestration is a major element in the pathophysiology of severe falciparum malaria [145,146].

### 9.1. A Possible Subdomain of the ER in Plasmodium for Host-Targeted Proteins

Export of proteins into the host erythrocyte begins in the ER. An ER domain, called the *Plasmodium* export compartment (PEC), that specializes in the export of proteins to the host erythrocyte has been proposed [147]. The PEC was initially identified due to the segregation of exported proteins into to a compartment distinct from but overlapping with the ER following brefeldin A treatment [148,149,150]. It appears that both PEXEL-containing exported proteins and PNEPs both accumulate in this compartment. Proteins with molecular masses of 68 kDa, 45 kDa, and 22 kDa were identified as possible resident proteins to this compartment [151]. These proteins partially co-localize with *Pf*Sar1 and *Pf*Sec31, which are components of coat protein complex II (COPII). COPII functions in the transport of vesicles from the ER to the Golgi [152]. In addition, the 68 kDa protein has been identified as *Pf*HSP70-2, an ER-resident protein [153]. These ER-resident proteins (*Pf*HSP70-2, *Pf*Sar1 and *Pf*Sec31) all exhibit a wider sub-cellular distribution than the compartment generated from exported proteins by brefeldin A. This suggests that exported proteins are found in a sub-compartment within the ER. The observations that PEXEL-containing and PNEPs are processed by different Sec61 translocons and proteases [113,116] are also consistent with distinct sub-compartments of the ER. The PEC is found adjacent to the parasite plasma membrane and may provide for a direct transit to the parasitophorous vacuole [147].

### 9.2. Plasmodium Has a Unique Translocon for Exporting Proteins from the Parasitophorous Vacuole

As discussed above, PNEPs may go directly from the ER to the PV, whereas PEXEL containing proteins possibly process through the Golgi before arriving in the PV (Figure 3). Thus, exported proteins destined for the erythrocyte also need to be translocated across the PVM after their arrival to the parasitophorous vacuole [132,154]. This translocation is accomplished by a large membrane complex called the *Plasmodium* translocon of exported proteins (PTEX) [154,155]. The three major proteins making up this complex are HSP101, PTEX150, and EXP2. PTEX is prepackaged into dense granules and transferred to the PVM after parasite invasion [156]. HSP101 is a chaperone that unfolds proteins and threads them through a pore formed from PTEX150 and EXP2. Another protein complex located in the PV, called exported protein interacting complex (EPIC), may participate in the movement of cargo within the PV and its delivery to PTEX [157]. Both PEXEL-containing effector proteins and PNEPs are translocated by PTEX [158,159]. Presumably, resident proteins of the parasitophorous vacuole are excluded from EPIC and PTEX.

Interestingly, EXP2 also functions as a nutrient channel for small metabolites when it is not associated with HSP101 and PTEX150 [160]. The PTEX channel for the movement of exported proteins from the parasitophorous vacuole to the host erythrocyte cytoplasm appears unique to *Plasmodium*. Homologs of EXP2 are found in *Toxoplasma* and other vacuole dwelling apicomplexans even though PTEX is not [161]. This suggests that EXP2 has been repurposed in *Plasmodium* for two transport related functions. Other differences between the endomembrane systems of *Plasmodium* and *Toxoplasma* have been noted (Table 3). Most of these differences are related to how the two parasites modify their host cells and may reflect differences between erythrocytes and nucleated cells as host cells. However, *Plasmodium* and *Toxoplasma*, the two most studied apicomplexans, exhibit more similarities in their endomembrane systems than differences.

### 9.3. Extraparasite Trafficking within the Erythrocyte Cytoplasm

**Figure 5:**
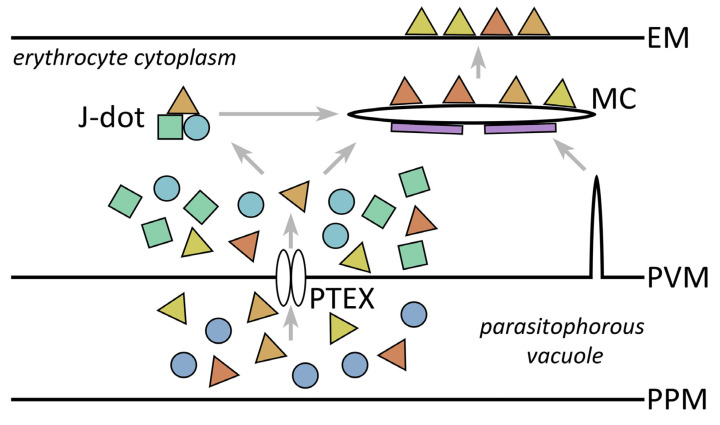
Possible components of extraparasite transport. After crossing the parasite plasma membrane (PPM) and arriving in the parasitophorous vacuole, proteins destined for the erythrocyte (triangles) are translocated across the parasitophorous vacuole membrane (PVM) via *Plasmodium* translocon of exported proteins (PTEX). Chaperones in the parasitophorous vacuole and the erythrocyte cytoplasm may assist in the process. HSP40 (circles) and HSP70-x (squares) interact with cargo proteins (triangles) to form J-dots. Cargo proteins may be moved from J-dots to the Maurer’s clefts (MC). Most Maurer’s cleft resident proteins (rectangles) are exported into the erythrocyte cytoplasm via PTEX. Cargo proteins are subsequently translocated from the Maurer’s clefts to the erythrocyte membrane (EM). The lipid components and possibly some resident proteins of the Maurer’s cleft may originate from the PVM.

The mature erythrocyte being devoid of endomembranes raises questions about how proteins are specifically targeted to the erythrocyte membrane and other specific locations within the infected erythrocyte. The parasite does not simply secrete proteins into the host erythrocyte but appears to generate a mechanism of extraparasite trafficking within the host erythrocyte. Molecular chaperones likely play a major role in this extraparasite trafficking. The parasite exports several chaperones into the parasitophorous vacuole and host erythrocyte cytoplasm, and these chaperones mediate many aspects of protein trafficking in *Plasmodium* [163,164]. For example, a paralog of HSP70, called *Pf*HSP70-x, and several HSP40 paralogs are exported into the parasitophorous vacuole and the host erythrocyte. Presumably, these chaperones assist in the refolding of exported proteins as they emerge from PTEX and may also assist in the association of exported proteins with the TVN, Maurer’s clefts, or the erythrocyte membrane. These various membranous structures may also participate in the movement of parasite proteins to the erythrocyte membrane. For example, two potential intermediate compartments in the transport of parasite proteins to the erythrocyte membrane are J-dots and Maurer’s clefts (Figure 5).

J-dots are highly mobile large molecular complexes found in the cytoplasm of infected erythrocytes consisting of an exported HSP40 [165] and *Pf*HSP70-x [166]. The HSP40 binds to cholesterol as well as *Pf*HSP70-x, and thus, J-dots may have a membranous nature [167]. Furthermore, the adhesin *Pf*EMP1 and other exported proteins are transiently associated with J-dots implying a role in extra-parasite trafficking [136,164,165]. Maurer’s clefts are membrane bound structures found in the host erythrocyte cytoplasm of infected cells [142]. Initially, the Maurer’s clefts are mobile within the cytoplasm of the erythrocyte but as the parasite matures, they become tethered to the erythrocyte membrane [168]. It has been proposed that cargo proteins, such as *Pf*EMP1, may move from the J-dots to the Maurer’s clefts [136,169].

Several proteins associated with Maurer’s clefts have been identified [136,170]. Some of these proteins play a role in the shape of the clefts. Other proteins are involved in loading erythrocyte membrane bound cargo onto the clefts, and other proteins are involved in transferring the cargo from the clefts to the erythrocyte membrane. The primary cargo of the Maurer’s clefts appears to be the adhesin *Pf*EMP1 [171]. Accordingly, many of the Maurer’s clefts proteins seem to be limited to the subgenus *Laverania* which includes *P. falciparum* and parasites of the great apes [172]. As discussed above, the increased virulence associated with *P. falciparum* is correlated with the expression of the *Pf*EMP1 adhesin on the erythrocyte surface. In addition, *P. falciparum* exports many more proteins into the host erythrocyte than other *Plasmodium* species [173]. Nonetheless, orthologs for some Maurer’s clefts proteins are found in rodent parasites and the *P. falciparum* proteins function in transgenic rodent parasites [174]. These results imply that the general features of transporting and translocating parasite proteins to the erythrocyte membrane are conserved in *Plasmodium*.

The origins of J-dots and Maurer’s clefts, and the relations of these structures to the membranous loops and whorls that extend from the PVM, are not known [84]. No stable continuities between Maurer’s clefts and either the PVM or the host erythrocyte membrane have been noted [142]. However, recently, a Maurer’s cleft protein, call skeleton-binding protein 1 (SBP1), and electron dense material associated with Maurer’s clefts have been observed to originate in the parasite and pass through the parasitophorous vacuole in route to the mature Maurer’s clefts [175]. Furthermore, Maurer’s clefts adjacent to the PVM suggest that Maurer’s clefts are derived from the PVM.

## 10. Summary

Endocytosis and exocytosis are hallmark features of eukaryotes, including pathogenic eukaryotic microbes. Secretory processes are often directly linked with pathogenesis and thus can be viewed as virulence factors. Virulence factors associated with secretory processes in these species are primarily adhesins expressed on the surface of the parasites and secreted proteins. Many of the secreted proteins are proteases that damage host tissue and allow parasite invasion of tissue. Other virulence factors are associated with the evasion of the immune system. In general, the targeting of proteins to the cell surface and the secretion of proteins into the host exhibit similar features as other eukaryotes. For the most part, protozoa have an endomembrane trafficking system consisting of the ER, Golgi, and endosomes that is comparable to other eukaryotes.

However, there does appear to be a reduction in the relative amount of Golgi and its function in the pathogenic protozoa. This is most pronounced in *Giardia* and *Plasmodium*. In *Giardia* most of the Golgi functions are found in the ER and it has been proposed that the parasite has completely lost the Golgi. Like *Giardia*, many Golgi functions are also found in the ER of *Plasmodium*. A distinct Golgi has been identified in *Plasmodium*, but it is rather limited in size and its exact role in membrane trafficking is not clear. In the other pathogenic protozoa, a distinct Golgi has been identified. However, quite often the Golgi is limited to a single stack, or possibly two, per cell. The position of the Golgi is polarized within the cell in the kineoplastids and is located near the flagellar pocket which is the site of all endocytic and exocytic activities. A specialized region of the ER is associated with the Golgi of trypanosomes. *Plasmodium* also appears to have sub-domains of the ER. It appears that decreased functionality of the Golgi and increased functions in the ER may be common feature in pathogenic protozoa.

Little is known about the endosomes of pathogenic protozoa. *Giardia* has a unique compartment with both endosome and lysosome like properties called peripheral vacuoles which also participates in the trafficking of proteins to the plasma membrane. Some repurposing of the endosomal compartment may also have occurred in the Apicomplexa as endosomes appear to be involved in the genesis of the apical organelles.

The Apicomplexa have several unique endomembrane systems that are involved in host-pathogen interactions. Most notable are the highly specialized apical organelles that play a key role in invasion of host cells. These organelles include micronemes and rhoptries which participate in the formation of a junction between the parasite and host cell. This junction is linked to a unique actino-myosin motor complex called the glideosome. Their evolutionary origin likely relates to the attachment of free-living predatory protozoa to their prey. Over time these organelles may have evolved to facilitate interactions with host cells during the evolution of parasitism. For example, linkage of the host-parasite junction with the glideosome would allow for invasion. The glideosome is also associated with another unique compartment in Apicomplexa called the inner membrane complex.

After gaining entry into the host cell, apicomplexan parasites—especially the malaria parasite—extensively modify their host cells. The elaborate modifications of the host erythrocyte by the malaria parasite play an essential role in the parasite’s biology, as well as in the pathophysiology of the disease. These modifications involve the export of numerous parasite proteins into the host erythrocyte. Many of the exported proteins utilize a unique targeting sequence called PEXEL. PEXEL is also found in other apicomplexans and pathogenic stramenopiles. However, the number of PEXEL containing proteins appears to be greatest in *Plasmodium*, and among the malaria parasites, *P. falciparum* appears to have the largest repertoire of PEXEL-containing proteins. Coincidently, *P. falciparum* is the most virulent malaria parasite and the extensive export of material into the host erythrocyte contributes to this increased virulence. *Plasmodium* also has a unique translocon for the movement of proteins from the parasitophorous vacuole to the erythrocyte cytoplasm. Furthermore, the malaria parasite has created mechanisms to traffic proteins beyond its own plasma membrane within the cytoplasm of the erythrocyte.

## Figures and Tables

**Table 1:** Endomembranes of Major Protozoan Pathogens of Humans

Pathogen (Super Group)	Golgi	Microbodies	Unique Compartments
*Giardia* (Excavata)	No stacks and reduced function [13]	Peroxisomes [14]	Peripheral vacuoles (lysosome-like) [15] Mitosome ^2^ [16]
*Trichomonas* (Excavata)	Stacked [17]	lacking	Hydrogenosome ^2^ [16]
*Entamoeba* (Amorphea)	Vesicles [18]	lacking	Mitosome ^2^ [16]
Kinetoplastids ^1^ (Excavata)	Usually a single stack [19]	Glycosomes [20]	Flagellar pocket [21] Predominance of GPI-anchored surface molecules [19]
*Cryptosporidium* (TSAR)	Not yet identified	lacking	Apical organelles [22] Inner membrane complex [23]
*Toxoplasma* (TSAR)	Single stacked of 3-5 cisternae [24]	Peroxisomes (lipid metabolism) [25]	Apical organelles [22] Inner membrane complex [23] Apicoplast ^2^ [26]
*Plasmodium* (TSAR)	Single cisterna in blood stage [27]; single Golgi with 1–3 cisternae in mosquito stage [28]	lacking	Apical organelles [22] Inner membrane complex [23] Apicoplast ^2^ [26] Food vacuole (lysosome-like) [29]

^1^ Includes African trypanosomes, *Trypanosoma cruzi*, and *Leishmania***.** ^2^ Mitosomes, hydrogenosomes, and apicoplasts are not part of the endomembrane system and are included here to highlight unique features in protozoa.

**Table 2:** Apical Organelles.

Organelle	Description	Features
Microneme	Oval vesicles congregated at apical end	Contents include adhesins that are integrated into the microneme membrane. Secretion of microneme exposes the adhesin on surface of parasite at apical end. The adhesins bind to receptors on host cells to form a junction.
Rhoptry	Club-shaped organelles with duct at apical end.	Proteins found in the neck region of the rhoptry participate in the formation of the moving junction and glideosome. Material in bulbs of the rhoptry contributes to the formation of the parasitophorous vacuolar membrane.
Dense Granules	Secretory vesicles found at the apical end and throughout the parasite cytoplasm.	Material in the dense granules is released shortly after parasite invasion and modify the parasitophorous vacuole and host cell. Some species produce dense granules throughout the intracellular period.

**Table 3:** Differences Between Plasmodium and Toxoplasma.

Feature	*Plasmodium*	*Toxoplasma*
Replication	Schizogony (multiple rounds of nuclear replication followed by cytoplasmic segmentation)	Endodyogeny (internal formation of invasive stages)
IMC	De novo formation of IMC during each replication cycle	Extensive recycling of IMC material from mother cell to daughter cell
Dense Granules	Only found in invasive stages and contents released shortly after invasion	Found in invasive stages and continuously produced during intracellular period
PEXEL processing	Occurs in ER	Occurs in Golgi
PEXEL targeting	Primarily to the parasitophorous vacuole with possible exception of RESA to the dense granules	To the dense granules first and then to the parasitophorous vacuole
PVM translocon	PTEX	MYR1 [162]
